# Resveratrol inhibits IL-33–mediated mast cell activation by targeting the MK2/3–PI3K/Akt axis

**DOI:** 10.1038/s41598-019-54878-5

**Published:** 2019-12-05

**Authors:** Shotaro Nakajima, Kayoko Ishimaru, Anna Kobayashi, Guannan Yu, Yuki Nakamura, Kyoko Oh-oka, Katsue Suzuki-Inoue, Koji Kono, Atsuhito Nakao

**Affiliations:** 1 Department of Immunology, Faculty of Medicine, University of Yamanashi, Yamanashi, Japan; 2 Department of Pediatrics, Faculty of Medicine, University of Yamanashi, Yamanashi, Japan; 3 Department of Clinical and Laboratory Medicine, Faculty of Medicine, University of Yamanashi, Yamanashi, Japan; 4 Department of Immunology, Faculty of Medicine, Tukuba Advanced Research Alliance (TARA), University of Tsukuba, Tsukuba, Japan; 5 Department of Gastrointestinal Tract Surgery, Faculty of Medicine, Fukushima Medical University, Fukushima, Japan; 6 Department of Progressive DOHaD Research, Faculty of Medicine, Fukushima Medical University, Fukushima, Japan; 7 Present Address: Department of Progressive DOHaD Research, Department of Gastrointestinal Tract Surgery, School of Medicine, Fukushima Medical University, 1 Hikariga-oka, Fukushima City, Fukushima 960-1295 Japan

**Keywords:** Basophils, Mast cells

## Abstract

Interleukin-33 (IL-33)/ST2–mediated mast cell activation plays important roles in the pathophysiology of allergic diseases. Hence, pharmacologically targeting the IL-33/ST2 pathway in mast cells could help to treat such diseases. We found that resveratrol inhibits IL-33/ST2–mediated mast cell activation. Resveratrol suppressed IL-33–induced IL-6, IL-13, and TNF-α production in mouse bone marrow–derived mast cells (BMMCs), mouse fetal skin–derived mast cells, and human basophils. Resveratrol also attenuated cytokine expression induced by intranasal administration of IL-33 in mouse lung. IL-33–mediated cytokine production in mast cells requires activation of the NF-κB and MAPK p38–MAPK-activated protein kinase-2/3 (MK2/3)–PI3K/Akt pathway, and resveratrol clearly inhibited IL-33–induced activation of the MK2/3–PI3K/Akt pathway, but not the NF-κB pathway, without affecting p38 in BMMCs. Importantly, resveratrol inhibited the kinase activity of MK2, and an MK2/3 inhibitor recapitulated the suppressive effects of resveratrol. Resveratrol and an MK2/3 inhibitor also inhibited IgE-dependent degranulation and cytokine production in BMMCs, concomitant with suppression of the MK2/3–PI3K/Akt pathway. These findings indicate that resveratrol inhibits both IL-33/ST2–mediated and IgE-dependent mast cell activation principally by targeting the MK2/3–PI3K/Akt axis downstream of p38. Thus, resveratrol may have potential for the prevention and treatment of broad ranges of allergic diseases.

## Introduction

IL-33, a member of the IL-1 family, plays multiple roles in tissue homeostasis and inflammation via its receptor ST2. Recent studies suggest that the IL-33/ST2 interaction plays a critical role in the development of allergic diseases. Upon exposure to allergens, epithelial cells release IL-33 as an alarmin, which activates ST2–expressing immune cells including mast cells and type 2 innate lymphoid cells (ILC2), leading to the production of IL-5 and IL-13 and initiation of allergic inflammation. The IL-33/ST2 pathway is thought to contribute to the development of both IgE–dependent and -independent allergic inflammation^1^. Therefore, targeting IL-33/ST2 signaling in mast cells (or ILC2) represents an important strategy for the prevention and treatment of broad ranges of allergic diseases.

Resveratrol (trans-3,4,5-trihydroxystilbene) is a natural polyphenol produced by several flowering plants, such as red grapes, in response to environmental stresses. Resveratrol has pleiotropic effects on many cell types, thereby exerting beneficial effects on aging, metabolism, cancer, and inflammation^2,3^. In mast cells, resveratrol limits IgE-mediated degranulation, prostaglandin D_2_ synthesis, and cytokine production, in association with inhibition or activation of multiple pathways including phospholipase C (PLC)γ, extracellular-signal-regulated kinase (ERK)/ c-Jun NH_2_-terminal kinase (JNK)/p38, AMP-activated protein kinase (AMPK)/Sirt1, and nuclear factor-κB (NF-κB)^4,5^. However, it remains unclear whether resveratrol inhibits IL-33/ST2–mediated mast cell activation.

IL-33/ST2–mediated cytokine production in mast cells requires activation of the NF-κB and mitogen-activated protein kinase (MAPK) p38 pathways^6^. MAPK-activated protein kinases 2 and 3 (MK2 and MK3), serine/threonine kinases downstream of p38, are pivotal for IL-33/ST2–induced mast cell activation. Bone marrow–derived mast cells (BMMCs) produce IL-6, IL-13, and tumor necrosis factor (TNF)-α upon IL-33 stimulation, and this response depends on MK2/3-mediated activation of the phosphatidylinositol 3-kinase (PI3K)/Akt pathways^7^.

In this study, we sought to determine whether resveratrol can inhibit IL-33/ST2–mediated mast cell activation, and if so, how. For this purpose, we examined the effects of resveratrol on IL-33–mediated IL-6, IL-13, and TNF-α production and multiple signaling pathways, including MK2/3–PI3K/Akt, in BMMCs. We also examined the effects of resveratrol on IgE–dependent degranulation and cytokine production in BMMCs. The results identify resveratrol as an inhibitor for IL-33/ST2–mediated mast cell activation and MK2/3 as a novel target of resveratrol.

## Materials and Methods

### Materials

Reagents used in this study were acquired from the indicated suppliers: trans-resveratrol, cyclodextrin, 5Z-7-Oxozeaenol and Evans blue (Sigma-Aldrich, St. Louis, MO, USA); LY294002, wortmannin, and ICI 182,780 (Abcam, Cambridge, UK); PF-3644022 (TOCRIS Bioscience, Bristol, U.K.); recombinant mouse IL-3, recombinant mouse stem cell factor (SCF), and recombinant human IL-33 (PeproTech, Rocky Hill, NJ, USA); recombinant human IL-3 (Thermo Fisher Scientific, Wilmington, DE, USA); recombinant mouse IL-33 (R & D systems, Minneapolis, MN, USA); anti-TNP IgE, anti–DNP mouse IgE mAb, anti–mouse CD16/32, PE–conjugated anti–mouse c-kit Ab, and APC–conjugated anti–mouse ST2 Ab (BD Bioscience, San Jose, CA, USA); DNP–BSA (Cosmo Bio, Tokyo, Japan); APC–conjugated anti–mouse CD63 Ab (Miltenyi Biotec, Bergisch Gladbach, Germany); anti–phospho–transforming growth factor β-activated kinase 1 (TAK1) Ab (Thr184/187; #4508), anti–phospho–IκB kinase (IKK) α/β Ab (Ser176/177; #2078), anti–phospho–p65 Ab (Ser536; #3033), anti–phospho–p38 Ab (Thr180/Thy182; #4511), anti–phospho–MK2 Ab (Thr334; #3007), anti–phospho–Akt Ab (Ser473; #4060), anti–phospho–Gab2 Ab (Tyr452; #3882), anti–phospho–Syk Ab (Tyr525/526; #2710), anti–phospho–p70S6K Ab (Tyr389; #9234), anti–phospho–AMPK Ab (Thr172; #2535), anti–β-actin Ab (#4970), and AICAR (#9944) (Cell Signaling Technology, Danvers, MA, USA).

### Mice

Male 6–8-week-old C57BL/6 mice were purchased from Japan SLC (Tokyo, Japan) and housed under 12-hour light/12-hour dark conditions. Mice were orally treated with 10 mg/kg resveratrol or 2% cyclodextrin in phosphate-buffered saline (PBS) as a control. After 2 hours, the mice were intranasally challenged with recombinant mouse IL-33 for 24 hours. mRNA levels of IL-6 and IL-13 in lung were assayed by qPCR. All animal experiments were approved by the Institutional Review Board of the University of Yamanashi and carried out according to the guidelines.

### Generation of mouse BMMCs and fetal skin-derived mast cells (FSMCs)

BMMCs were prepared from femoral bone marrow cell suspensions from male C57BL/6 mice, as described previously^8^. Briefly, crude bone marrow cells were cultured in RPMI 1640 supplemented with 10% fetal bovine serum, 2 mM L-glutamine, 10 mM nonessential amino acids, penicillin/streptomycin, 10 mM sodium pyruvate, and 50 μM 2-ME (complete RPMI 1640) in the presence of 10 ng/ml recombinant mouse IL-3 (rmIL-3). Floating cells were refreshed twice per week, and further expanded for 2–4 weeks in fresh complete RPMI 1640 supplied with murine rIL-3. Finally, the cells (>95% FcεR1^+^c-kit^+^) were used as BMMCs without further purification. FSMCs were generated from fetal skin of C57BL/6 mice on day 14, as described previously^9^. Briefly, single-cell suspensions were prepared from excised trunk skin specimens by limited trypsinization in complete RPMI 1640 for 20 minutes at 37 °C. Crude cells were cultured in complete RPMI 1640 in the presence of 10 ng/ml rmIL-3 and 10 ng/ml recombinant mouse SCF. Nonadherent and loosely-adherent cells were collected 2 weeks after the original culture and were enriched for mast cells by Percoll density-gradient centrifugation. Finally, the cells (>95% FceR1^+^c-kit^+^) were used as FSMCs.

### Isolation of human basophils from peripheral blood mononuclear cells (PBMCs)

The use of buffy coats from healthy donors was approved by the ethical committees in University of Yamanashi and carried out according to the guidelines, and written informed consent was provided according to the Declaration of Helsinki. PBMCs were isolated from buffy coats using Ficoll–Paque (GE Healthcare, Little Chalfont, U.K.). Human basophils were isolated using the MACS magnetic cell separation system (Basophil Isolation Kit II, human; Miltenyi Biotec). Isolated human basophils were cultured in complete RPMI 1640 supplemented with 10 ng/ml recombinant human IL-3 for 18 hours, and then treated with 100 ng/ml recombinant human IL-33 for 6 hours in the absence or presence of 25 μM resveratrol.

### Flow cytometry analysis

BMMCs were stained with antibodies specific for PE-conjugated mouse c-kit, APC-conjugated mouse FcεRI, APC–conjugated mouse ST2, or APC–conjugated mouse CD63, respectively, in the presence of rat anti-mouse antibodies against CD16/32. After washing with PBS, the stained cells were analyzed on a BD Accuri C6 flow cytometer (BD Biosciences). For detection of apoptotic cells, BMMCs were incubated with 5 μl PE–conjugated annexin V and 5 μl 7-AAD in 100 μl of 1×binding buffer for 15 min. After adding 400 μ of 1×binding buffer, the stained cells were analyzed on the flow cytometer. Flow cytometry data were analyzed using the CellQuest software (BD Biosciences).

### Cell proliferation assay

Cell proliferation was monitored using the Cell Counting Kit-8 (Dojindo Laboratory, Kumamoto, Japan).

### Quantitative real-time PCR

Total RNA was isolated from BMMCs and mice lungs using the RNeasy Mini Kit (QIAGEN, Valencia, CA, USA). RNA was quantified on a NanoDrop ND-1000 spectrophotometer (Thermo Fisher Scientific). Complementary DNA (cDNA) was synthesized using the PrimeScript RT-PCR Kit (Takara, Ootsu, Japan). Quantitative real-time PCR (qPCR) was performed on a 7300 Fast Real-Time PCR System (Applied Biosystems, Carlsbad, CA, USA) using qPCR Master Mix (Applied Biosystems) with specific primers and probes against mouse IL-6, IL-13, TNF, and GAPDH (Applied Biosystems). qPCR data were normalized against the corresponding levels of GAPDH mRNA.

### Chromatin immunoprecipitation (ChIP) assay

ChIP assay was performed using the SimpleChIP Enzymatic Chromatin IP Kit (Agarose Beads) (Cell Signaling Technology; #9002). BMMCs were cross-linked in 1% formaldehyde prior to ChIP. ChIP-grade anti–mouse p65 Ab (Cell Signaling Technology) and purified rabbit IgG were used for immunoprecipitation. Immunoprecipitated DNA and input were analyzed by qPCR using primers and TaqMan probes against the promoter region of the TNF-α. Primers used were as follows: mouse TNF-α κB2 promoter: sense, 5′-GCTCATGATCAGAGTGAAAGGAGAA-3′, antisense, 5′-GGAATGAACTCAGCCCTGGG-3′, TaqMan probe, 5′-FAM-CTTGTGAGGTCCGTGAATT-NFQ-3′; mouse TNF-α κB3 promoter: sense, 5′-CCTTCAGCCACTTCCTCCAA-3′, antisense, 5′-TCTGAAAGCTGGGTGCATAAGG-3′, TaqMan probe, 5′-FAM-AAGCCCCCTGTTTGAGTTC-NFQ-3′ (Applied Biosystems).

### Enzyme-linked immunosorbent assay (ELISA)

The concentrations of IL-6, IL-13, TNF-α, and histamine in supernatants of cell cultures were determined by ELISA. Kits for mouse IL-6 and TNF-α (eBioscience), mouse histamine (Oxford Biomedical Research, Rochester Hills, MI), mouse IL-13, and human IL-13 (R & D systems) were obtained from the indicated suppliers.

### *In vitro* p38 and MK2 kinase assay

*In vitro* kinase assay of p38 and MK2 was performed using the CycLex p38 Kinase Assay/Inhibitor Screening Kit and CycLex MK2 Kinase Assay/Inhibitor Screening Kit (CycLex, Nagano, Japan). The inhibitory effect of resveratrol on p38 and MK2 activity was evaluated by direct addition of resveratrol to the p38 and MK2 positive controls (CycLex).

### β-Hexosaminidase release assay

BMMCs were incubated with 1 μg/ml anti–DNP mouse IgE mAb for 1 hour at 4 °C, and then stimulated with 1 μg/ml of anti-mouse IgE antibody with or without resveratrol for 40 minutes at 37 °C. Total release was obtained by adding 1% Triton buffer for 40 min. The supernatants were collected from each well and mixed with *p*-nitrophenyl-*N*-acetyl-β-D-glucosaminide to determine the enzymatic activity of the released β-hexosaminidase. After 90 min at 37 °C, the reaction was stopped by adding 0.2 M glycine solution, and measured by absorption spectrometer (at 405 nm). The percentage of β-hexosaminidase release was calculated as follows:

% release = (OD of stimulated supernatant – OD of unstimulated supernatant)×100/(OD of supernatant of Triton-lysed cells – OD of unstimulated supernatant)

### Western blot analysis

BMMCs were lysed in RIPA buffer (25 mM Tris-HCl, pH7.6, 150 mM NaCl, 1% Triton X100, 1% sodium deoxycholate, 0.1% SDS) with protease inhibitor cocktail (Merck Millipore, Burlington, MA) and vanadate (FUJIFILM Wako Pure Chemical Corporation, Osaka, Japan). Cell lysate was dissolved in sample buffer containing 50 mM dithiothreitol and bromophenol blue, and then boiled for 5 min. Protein concentrations were measured on a NanoDrop ND-1000 (Thermo Fisher Scientific). Proteins were subjected to SDS-PAGE gels and transferred to polyvinylidene fluoride membranes. Blots were immersed in 5% milk blocking solution for 1 hour at room temperature (RT), followed by incubation with primary antibody solution overnight at 4 °C. Membranes were washed three times with TBS/T, and then incubated in a secondary antibody solution for 2 hours at RT. Immunoreactive proteins were visualized using ECL Prime (GE Healthcare).

### Reporter assays

Using the Mouse Macrophage Nucleofector kit (VPA-1009; Lonza, Basel, Switzerland), BMMCs were transiently transfected with the pNFκB-Luc reporter plasmid, with pRL-CMV as an internal control. After 48 hours, the transfected BMMCs were stimulated with IL-33 in the absence or presence of resveratrol. Relative NF-κB luciferase activity was normalized against transfection efficiency.

### Measurement of Ca^2+^ mobilization

Intracellular Ca^2+^ mobilization was analyzed using Fluo-3 AM (Dojindo, Kumamoto, Japan). Briefly, IgE–sensitized BMMCs were loaded with 4 μM Fluo-3 AM for 30 minutes at 37 °C. The cells were then resuspended in fresh medium and stimulated with antigen. Changes of dye fluorescence in BMMCs were monitored by flow cytometry immediately after antigen stimulation. In figures, Ca^2+^ mobilization is represented as relative fluorescence intensity.

### Passive cutaneous anaphylaxis

Mouse anti-TNP IgE (100 ng) was intradermally injected into dorsal skin. After 24 hours, the mice were challenged by intravenous injection with 50 μg of DNP-BSA in PBS containing 0.2% Evans blue dye. Vascular permeability was visualized 40 minutes later as blue staining of the injection areas on the inside of the skin. These staining sites were digitized using a high-resolution color camera, and the images were analyzed using ImageJ 1.43 (NIH, Bethesda, MD, USA). Briefly, the color-scale images (the upper panels) were converted for hue/saturation/brightness stack type images, and then the hue/saturation/brightness stack image was split into hue, saturation and brightness images respectively. Only blue-stained were selected from the hue image by using the threshold tool. These images were then combined with the saturation image, and the density values for the blue-stained areas were measured using analyze tool^10^.

### Statistical analysis

The statistical analyses were performed using the unpaired Student’s *t*-test for two group-comparisons. For multigroup comparisons, we applied one-way ANOVA with post hoc testing using Tukey-Kramer multiple comparisons. A value of *p* < 0.05 was considered to be significant.

## Results

### Resveratrol inhibits IL-33–induced mast cell activation

First, we assessed the cytotoxicity of resveratrol in mast cells. Treatment of mouse bone marrow–derived mast cells (BMMCs) with resveratrol for 6 hour at a concentration of 10–100 μM did not affect cell viability as judged by Annexin V staining (Fig. S1A), whereas concentrations of 50 and 100 μM, but not 10 and 25 μM, significantly decreased cell proliferation as judged by WST assay (Fig. S1B). These findings in WST assay are consistent with those of a previous study^11^. Therefore, resveratrol at the concentrations of 1–25 μM was used for subsequent *in vitro* studies.

**Figure 1:**
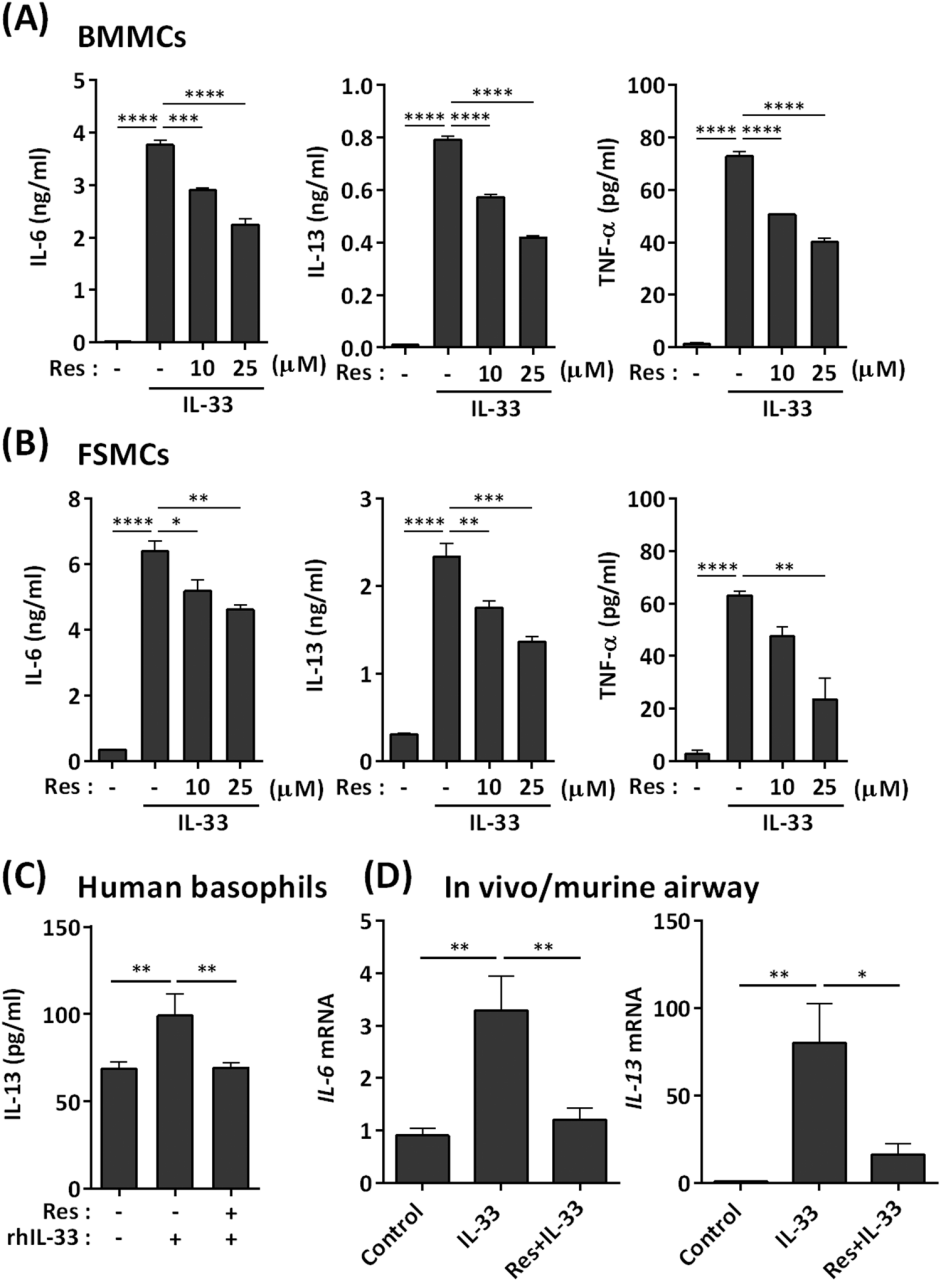
Inhibition of IL-33–induced mast cell activation by resveratrol. (**A,B**) ELISA of IL-6, IL-13, and TNF-α in culture supernatants of BMMCs (**A**) and FSMCs (**B**) treated with resveratrol and exposed to 1 ng/ml rmIL-33 for 6 h (n = 3). (**C**) ELISA of IL-13 in human peripheral blood basophils treated with resveratrol and exposed to rhIL-33 for 6 h in the presence of rhIL-3 (n = 3). (**D**) Mice were orally treated with resveratrol for 2 h, and then challenged with intranasal administration of 1 μg IL-33 for 24 h. mRNA expression of IL-6 and IL-13 in the airway was determined by qPCR (n = 4–6). *P < 0.05, **P < 0.01, ***P < 0.001, ****P < 0.0001.

As previously reported^12^, BMMCs produced IL-6, IL-13, and TNF-α in response to IL-33 (Fig. 1A). Pretreatment of BMMCs with resveratrol for 1 hour prior to IL-33 stimulation inhibited these responses in a dose-dependent manner (Fig. 1A). This suppression occurred at the transcriptional level (Fig. S1C). Fetal skin-derived mast cells (FSMCs) are mouse connective tissue–type skin-derived mast cells (Fig. S1D), and similar results were observed in FSMCs (Fig. 1B). In addition, resveratrol inhibited IL-33–induced IL-13 production in human basophils (Fig. 1C).

Intranasal administration of IL-33 to the mouse increased IL-6 and IL-13 expression in the lung, a response that is dependent on mast cells^13,14^. Oral administration of resveratrol significantly inhibited IL-33–induced IL-6 and IL-13 mRNA expression in mouse lungs (Fig. 1D). Thus, resveratrol inhibits IL-33–mediated mast cell activation both *in vitro* and *in vivo*.

### Resveratrol does not affect IL-33–mediated activation of the NF-κB pathway in BMMCs

**Figure 2:**
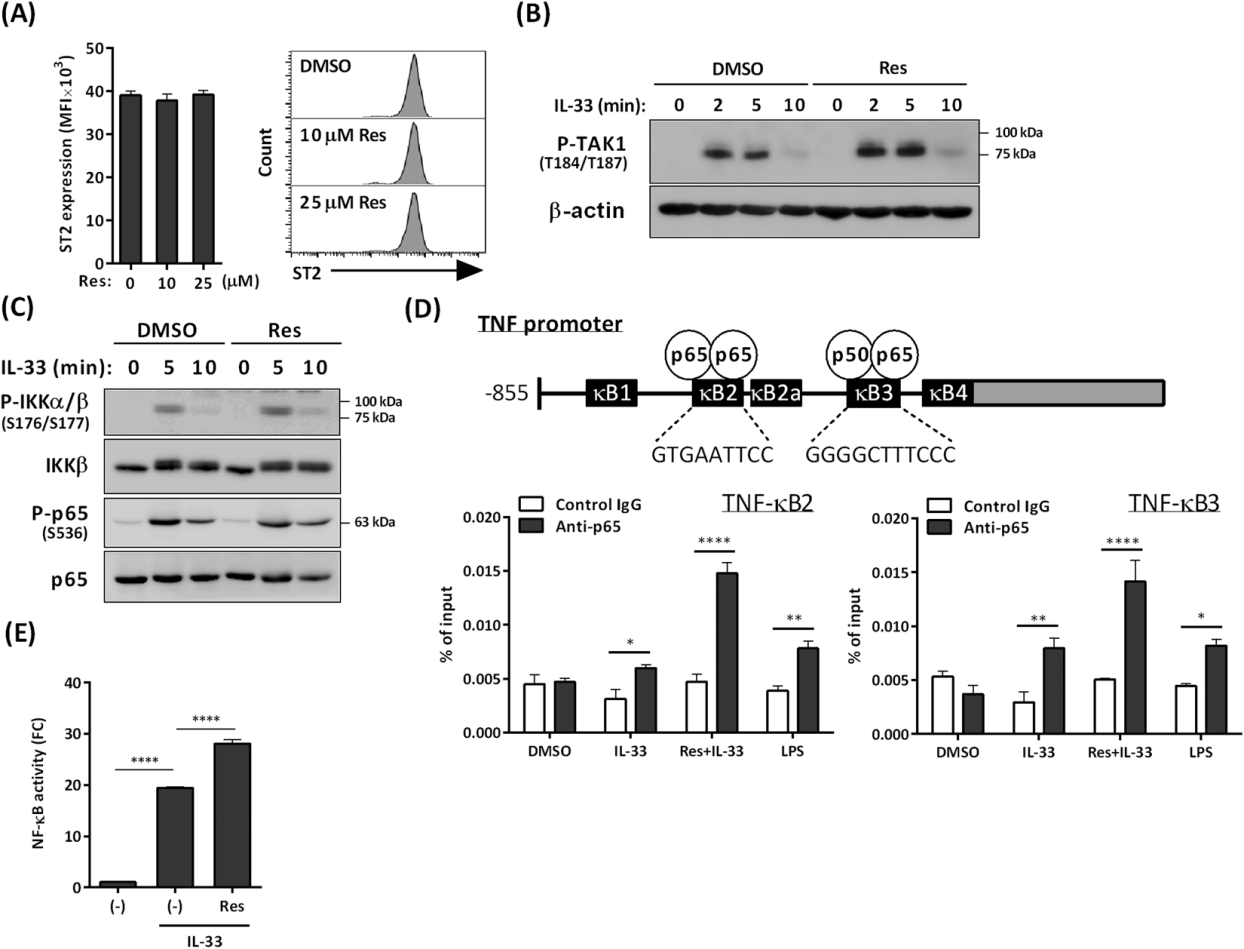
Effects of resveratrol on IL-33–mediated activation of NF-κB pathway in BMMCs. (**A**) Flow cytometry analysis of ST2 in BMMCs treated with or without resveratrol for 6 h (n = 3) (*left*), and representative histograms were shown (*right*). (**B,C**) Western blot analysis of phospho–TAK1 (B), phospho–IKKα/β, and phospho–p65 (**C**) in BMMCs stimulated with 1 ng/ml IL-33 for up to 10 min in the presence or absence of 25 μM resveratrol. (**D**) ChIP assay of p65 binding to the κB2 and κB3 regions of the TNF-α promoter in BMMCs treated with IL-33 or 1 μg/ml LPS in the presence or absence of resveratrol (n = 3). (**E**) Luciferase assay of NF-κB activity in BMMCs treated with IL-33 in the presence or absence of resveratrol (n = 3). *P < 0.05, **P < 0.01, ****P < 0.0001.

IL-33/ST2 interaction on mast cells activates TAK1, which leads to activation of the NF-κB and MAPK p38 pathways, leading to the production of cytokines including IL-6, IL-13, and TNF-α^15^. We found that resveratrol did not affect surface expression levels of ST2 or IL-33–induced phosphorylation of TAK1 in BMMCs (Fig. 2A,B). Hence, we first examined the effects of resveratrol on IL-33–induced activation of the NF-κB pathway.

Western blot analysis revealed that IL-33–induced phosphorylated IKKα/β and p65, a subunit of NF-κB in BMMCs, were not affected by resveratrol (Fig. 2C). Consistent with this, ChIP assays showed that resveratrol did not inhibit but rather enhanced IL-33–induced DNA binding of NF-κB to the promoter region of TNF-α (TNF-κB2: the sequence recognized by the p65–p65 homo dimer; TNF-κB3: NF-κB consensus sequence recognized by the p50–p65 heterodimer). In addition, a reporter assay also showed that resveratrol did not suppress, but instead enhanced, transcriptional activation of NF-κB in BMMCs (Fig. 2D,E).

Resveratrol modulates the inflammatory response, in association with inhibition of NF-κB pathway, by functioning as an estrogen receptor agonist^16^. However, ICI 182,780, an estrogen receptor antagonist, did not reverse the suppressive effects of resveratrol on IL-33–induced IL-13 and TNF-α mRNA expression (Fig. S2), suggesting that resveratrol does not trigger the estrogen receptor–mediated inhibition of NF-κB in BMMCs.

These results suggest that the NF-κB pathway is not involved in the suppressive effect of resveratrol on IL-33–mediated activation of BMMCs.

### Resveratrol inhibits IL-33–induced activation of MK2–PI3K–Akt pathway in BMMCs

Given that resveratrol does not inhibit IL-33–induced activation of TAK1 and NF-κB pathway in BMMCs, we next examined the effects of resveratrol on MAPK p38 pathways, in particular the p38–MK2/3–PI3K/Akt pathway, which is crucial for IL-33–mediated IL-6, IL-13, and TNF-α production in mast cells^7^.

**Figure 3:**
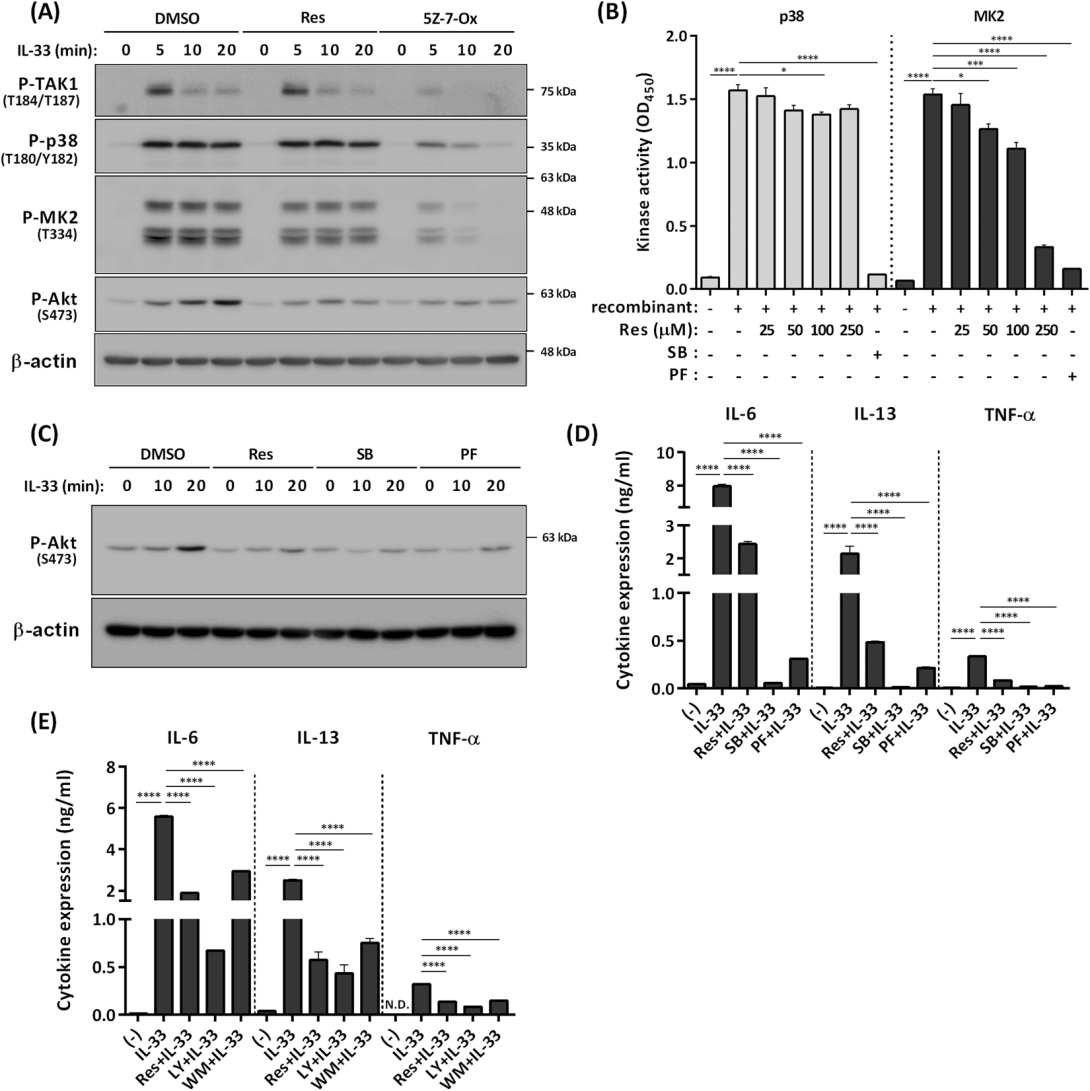
Inhibition of IL-33–induced mast cell activation via blockade of the MK2−PI3K−Akt pathway by resveratrol. (**A**) Western blot analysis of phospho–TAK1, phospho–p38, phospho–MK2, and phospho–Akt in BMMCs stimulated with 1 ng/ml IL-33 for up to 20 min in the presence or absence of 25 μM resveratrol or TAK1 inhibitor 5Z-7-Ox. The level of β-actin is shown at the bottom as a loading control. (**B**) *In vitro* p38 and MK2 kinase assay using p38 and MK2 positive controls (n = 3). SB: 10 μM SB203580, PF: 10 μM PF-3644022. (**C**) Western blot analysis of phospho–Akt in BMMCs stimulated with IL-33 for up to 20 min in the presence or absence of resveratrol, SB, or PF. The level of β-actin is shown at the bottom as a loading control. (**D**) ELISA of IL-6, IL-13, and TNF-α in BMMCs treated with IL-33 for 6 h in the presence or absence of resveratrol, 10 µM SB, or 10 μM PF (n = 3). (**E**) ELISA of IL-6, IL-13, and TNF-α in BMMCs treated with IL-33 for 6 h in the presence or absence of resveratrol, 5 μM LY294002 (LY), or 100 nM wortmannin (WM) (n = 3). *P < 0.05, ***P < 0.001, ****P < 0.0001; N.D., not detected.

Resveratrol did not affect IL-33–induced phosphorylation of p38 and MK2, but it significantly inhibited Akt activation (phosphorylation) (Fig. 3A). To examine whether resveratrol suppresses kinase activity of MK2 which is an upstream kinase of Akt in IL-33–mediated mast cell activation, we performed *in vitro* kinase assay using recombinant MK2 protein. *In vitro* kinase assay revealed that resveratrol dose-dependently and efficiently inhibited the kinase activity of MK2 compared with that of p38 (Fig. 3B), suggesting that MK2 activity might be a primary and direct target of resveratrol. Importantly, the MK2/3 specific inhibitor PF-3644022 recapitulated the suppressive activity of resveratrol on IL-33–induced mast cell activation: the drug inhibited IL-33–induced phosphorylation of Akt and suppressed IL-6, IL-13, and TNF-α production in BMMCs (Fig. 3C,D). As previously shown^7^, PI3K inhibitors LY294002 and wortmannin suppressed IL-33–induced IL-6, IL-13, and TNF-α production in BMMCs (Fig. 3E).

It should be noted that combination with resveratrol and PF-3644022 additively inhibited IL-33–induced expression of IL-13, but not IL-6 (Fig. S3), suggesting that resveratrol may inhibit IL-33–induced IL-13 production in BMMCs by targeting other molecules than MK2/3.

Frojdo *et al*. reported that resveratrol directly binds to p110α and p110β, the catalytic subunits of PI3K, and inhibits their activity *in vitro*^17^. To exclude the possibility that resveratrol directly inhibits PI3K activity in BMMCs, BMMCs were stimulated with stem cell factor (SCF) in the presence or absence of resveratrol; SCF activates the PI3K–Akt pathway via its receptor c-kit^18^. Resveratrol did not affect SCF-induced phosphorylation of Akt (Fig. S4), suggesting that resveratrol does not directly inhibit PI3K activity in BMMCs.

Collectively, these results suggest that resveratrol inhibits MK2 (and also possibly MK3) activity and subsequent activation of the PI3K/Akt pathway, leading to down-regulation of IL-6, IL-13, and TNF-α production in BMMCs, although it is still possible that resveratrol can target other molecules than MK2/3.

### Resveratrol also suppresses IgE–mediated mast cell activation via blockade of the MK2–PI3K–Akt axis

Because the PI3K/Akt pathway is critical for IgE–mediated mast cell activation^19^, and the roles of MK2/3 in IgE–mediated signaling in mast cells remain unknown, we examined the effects of resveratrol on IgE–dependent activation of BMMCs.

**Figure 4:**
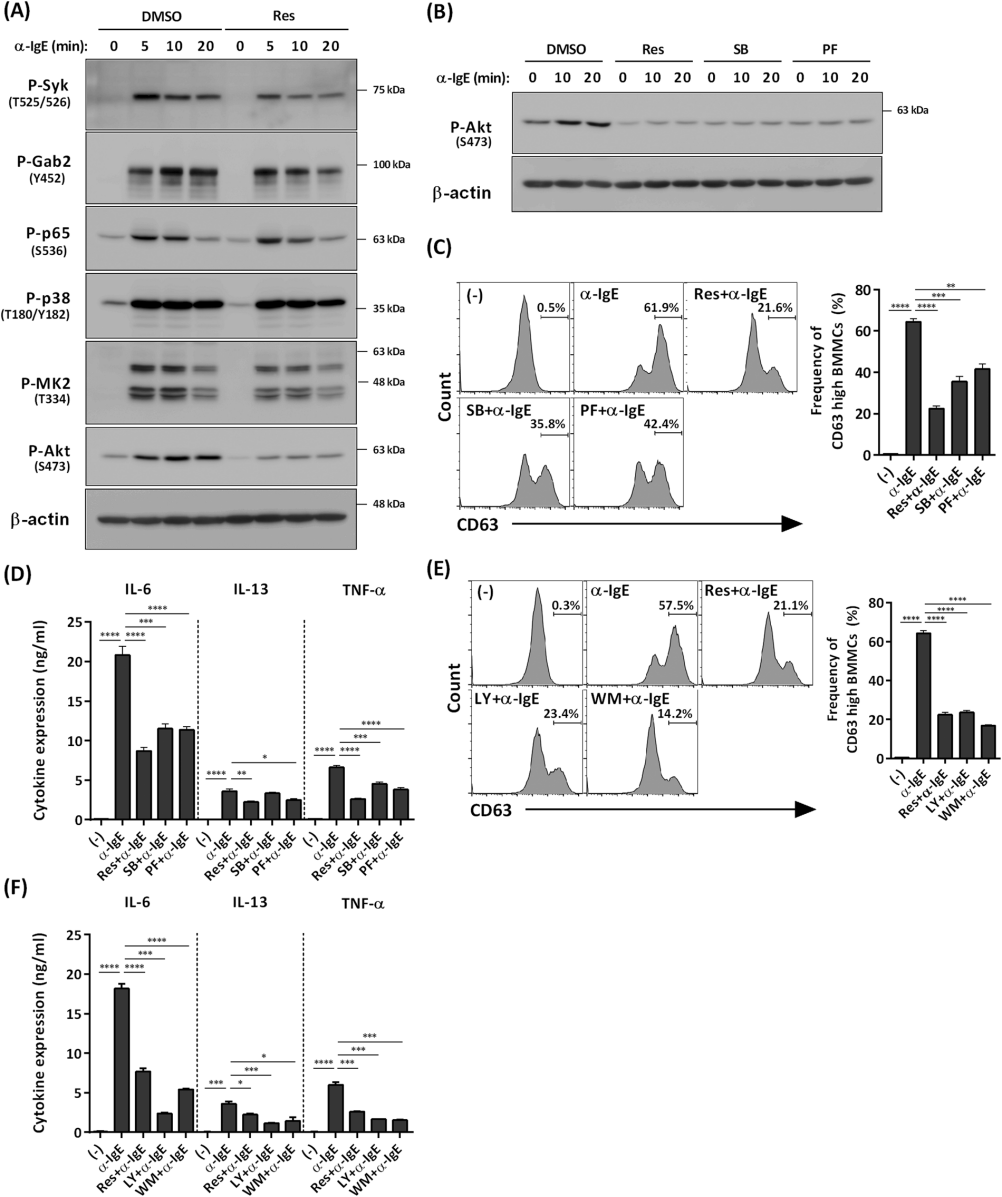
Suppression of IgE-mediated mast cell activation via inhibition of the MK2−PI3K−Akt axis by resveratrol. (**A**) Western blot analysis of phospho–Syk, phospho–Gab2, phospho–p65, phospho–p38, phospho–MK2, and phospho–Akt in BMMCs stimulated with antigen for up to 20 min in the presence or absence of 25 μM resveratrol. The level of β-actin is shown at the bottom as a loading control. (**B**) Western blot analysis of phospho–Akt in BMMCs stimulated with antigen for up to 20 min in the presence or absence of resveratrol, SB, or PF. The level of β-actin is shown at the bottom as a loading control. (**C**) Flow cytometry analysis of CD63 in BMMCs treated with antigen for 40 min in the presence or absence of resveratrol, 10 μM SB, or 10 μM PF (n = 3). (**D**) ELISA of IL-6, IL-13, and TNF-α in BMMCs treated with antigen for 6 h in the presence or absence of resveratrol, SB, or PF (n = 3). (**E**) Flow cytometry analysis of CD63 in BMMCs stimulated with antigen for 40 min in the presence or absence of resveratrol, 5 μM LY, or 100 nM WM (n = 3). (**F**) ELISA of IL-6, IL-13, and TNF-α in BMMCs treated with antigen for 6 h in the presence or absence of resveratrol, LY, or WM (n = 3). *P < 0.05, **P < 0.01, ***P < 0.001, ****P < 0.0001.

As previously reported^19,20^, IgE–mediated activation of mast cells induced phosphorylation of Syk, Gab2, p65, p38, and Akt. Moreover, IgE–mediated activation of BMMCs induced phosphorylation of MK2/3 (Fig. 4A), which has not been previously reported. Resveratrol slightly suppressed IgE–mediated phosphorylation of Syk, but did not affect IgE–dependent phosphorylation of Gab2, p65, p38 or MK2 (Fig. 4A). In contrast, resveratrol markedly inhibited IgE–dependent phosphorylation of Akt (Fig. 4A). Like SB203580, a p38 inhibitor, the MK2/3 inhibitor PF-3644022 significantly inhibited IgE–mediated phosphorylation of Akt (Fig. 4B), suggesting that MK2/3 might be one of the upstream kinases of Akt in IgE–mediated activation of BMMCs. SB203580 and PF-3644022 also blocked IgE–mediated degranulation (as judged by levels of the surface marker CD63), and IL-6, IL-13, and TNF-α production in BMMCs (Fig. 4C,D). We also confirmed that PI3K inhibitors LY294002 and wortmannin suppressed IgE–mediated degranulation and cytokine production in BMMCs (Fig. 4E,F).

Consistent with our findings and as previously reported^4^, resveratrol significantly suppressed IgE–dependent mast cell activation, as judged by calcium influx, degranulation, and cytokine production in BMMCs (Fig. S5A–D) and the PCA reaction in mice (Fig. S5E,F).

**Figure 5:**
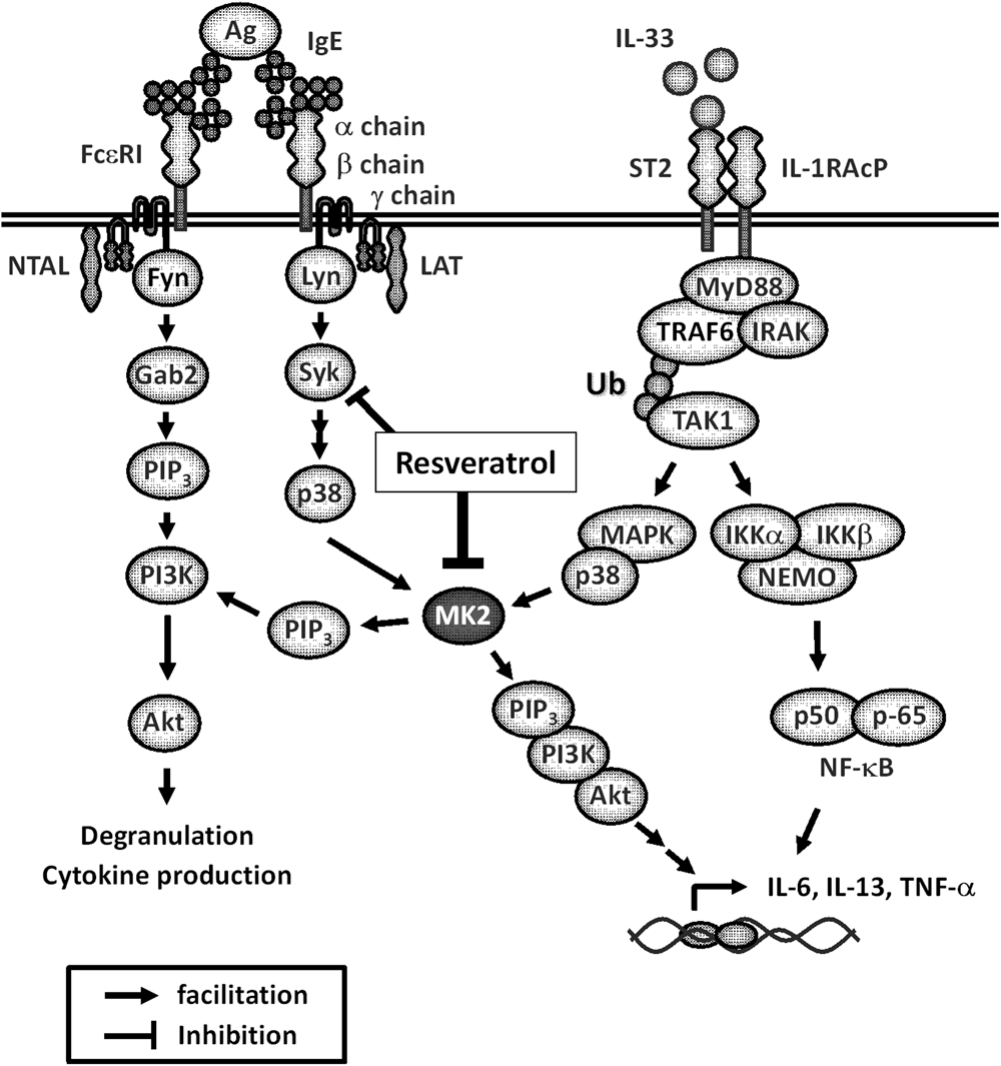
Mechanisms of the suppressive effect of resveratrol on IL-33– and IgE–mediated activation of mast cells. Resveratrol inhibits MK2, which plays important roles in mast cell activation induced by IgE–antigen and IL-33. Resveratrol also suppresses IgE–triggered phosphorylation of Syk slightly, which contributes to a greater potency of resveratrol for the inhibition of IgE–mediated activation of BMMCs.

These results suggest that inhibition of IgE–mediated activation of BMMCs by resveratrol can be attributed, at least in part, to the suppression of the MK2/3–PI3K/Akt pathway, as with the inhibition of IL-33–mediated activation of BMMCs (Fig. 5).

## Discussion

Recent studies have suggested that the IL-33/ST2 interaction plays critical roles in the development of allergic diseases. Hence, it would be clinically valuable to identify chemical compounds that interfere with IL-33/ST2 signaling in immune cells. We show here that resveratrol inhibits IL-33/ST2–mediated mast cell activation, as well as IgE–mediated activation. Furthermore, we identified MK2/3 as a possible target of resveratrol-mediated inhibition of mast cell activation. These findings implicate that resveratrol has potential utility for the prevention and treatment of broad ranges of allergic diseases.

Resveratrol clearly inhibits IL-33–dependent IL-6, IL-13, and TNF-α production in BMMCs, an immature type of mast cells, as well as in FSMCs, a mature connective type of mast cells. This finding is also relevant to basophils isolated from human peripheral blood, as well as to IL-33/mast cell–dependent lung inflammation in mice (Fig. 1). Thus, resveratrol can inhibit IL-33–dependent mast cell (and possibly also basophil activation) both *in vitro* and *in vivo*.

Resveratrol did not suppress ST2 expression or TAK1 activation. In addition, inhibition of IL-33–mediated IL-6, IL-13, and TNF-α production occurred at the transcription level. Therefore, it is likely that resveratrol inhibits activation of BMMCs by interfering with intracellular signaling pathways downstream of TAK1 and upstream of the transcriptional machinery.

IL-33–mediated cytokine production in mast cells requires activation of both the NF-κB pathway and p38–MK2/3–PI3K/Akt pathway. Unexpectedly, resveratrol did not affect, but instead enhanced, IL-33–induced activation of the NF-κB pathway. This conclusion is based on three different outputs: serine phosphorylation of IKK and p65, IL-33–induced DNA binding of NF-κB to the promoter region of TNF-α, and overall NF-κB transcriptional activation as determined by a reporter assay. These findings are somewhat surprising because most of the previous reports demonstrated that resveratrol markedly inhibited activation of the NF-κB pathway by several stimuli such as TNF-α and lipopolysaccharide^21–27^. On the other hand, several studies have reported that resveratrol stimulates NF-κB signaling in several cell types^28–30^. Thus, regulation of NF-κB activity by resveratrol likely depends on cell type and cellular contexts. The mechanisms how resveratrol enhances the TAK1-NF-κB pathway in BMMCs remains to be determined. Jaco *et al*. recently reported that activated–MK2 inhibited kinase activity of receptor-interacting kinase 1 (RIPK1), an upstream molecule of TAK1, to integrate cell survival and cytokine production^31^. RIPK1 is involved in regulations of not only TNFR signaling but also IL-1R signaling^32^. Therefore, MK2–mediated negative feedback loop via RIPK1 may be blocked by resveratrol, resulting in further activation of the TAK1-NF-κB pathway in BMMCs.

By contrast, resveratrol clearly inhibited IL-33–induced activation of the MK2/3–PI3K/Akt pathway without affecting activation of p38 in BMMCs. The MK2/3-PI3K–Akt–p70 ribosomal protein S6 kinase pathway is critical for induction of IL-6 and IL-13 by IL-33 in BMMCs and BMDCs^7,33^. Given that resveratrol inhibits the kinase activity of MK2, and an MK2/3 inhibitor recapitulated the suppressive effects of resveratrol on IL-33–induced activation of BMMCs, resveratrol suppresses production of IL-6, IL-13 and TNF-α principally by targeting the MK2/3–PI3K/Akt pathway in BMMCs. It remains to be determined how resveratrol inhibits the kinase activity of MK2/3.

Please note that we used a cell-free system to investigate the effect of resveratrol on MK2 kinase activity. Because the concentration of MK2 used in this system could be higher than the one in intact cells, higher doses of resveratrol might be needed when compared with the experiments using intact cells.

Our data suggested that resveratrol slightly suppressed IgE-mediated phosphorylation of Syk as previously reported (Fig. 4A)^4^. Because Syk is upstream of PI3K in mast cells^34^, de-phosphorylation of Syk might be also involved in the suppression of IgE–mediated Akt phosphorylation, degranulation, and cytokine production by resveratrol. The broad effects of resveratrol might contribute to strong inhibition for IgE-dependent activation of BMMCs.

There are several studies showing that resveratrol can inhibit IgE–mediated degranulation and cytokine production in association with the inhibition or activation of multiple pathways including PLCγ1, ERK/JNK/p38, Syk, NF-κB, and/or AMPK/Sirt1^4,5^. However, our results show that resveratrol does not inhibit phosphorylation of p38 or activation of the NF-κB pathway (Figs. 2 and 3). We also found that resveratrol (a subcytotoxic dose) did not affect phosphorylation of AMPK, regardless of the presence or absence of IgE stimulation in BMMCs, and AICAR, an AMPK activator, did not affect IgE-mediated Akt phosphorylation in BMMCs. These results suggest that resveratrol does not block IL-33– or IgE–mediated activation of BMMCs by targeting AMPK (Fig. S6). In addition, our results demonstrated that resveratrol clearly blocked IL-33– and IgE–mediated phosphorylation of Akt in BMMCs, whereas Shirley *et al*. reported that resveratrol did not inhibited IgE–dependent phosphorylation of Akt in human skin mast cells even if it suppressed release of allergic inflammatory mediators^35^. The discrepancy between the previous^4,5,35^ and present findings are unclear. Again, regulation of IgE-mediated signaling in mast cells by resveratrol may depend on the cell type (e.g., primary mast cells vs. mast cell lines, species differences [mice, rats, and humans]), cellular contexts (e.g. differentiation or activated status), and methods of stimulation and analysis (e.g. antigen vs FcεRI cross-linking, treatment duration, assay time, etc.).

Acute exposure of mast cells to IL-33 accelerates cytokine expression and FcεRI-triggered degranulation^36,37^. Based on its acute effects on mast cells (and type 2 innate lymphoid cells), IL-33 is thought to play a critical role in the pathogenesis of allergic disease. Conversely, long-term exposure to IL-33 reduces antigen-triggered mast cell activation as a feedback to chronic alarmin exposure^38–41^, suggesting that IL-33 may have a protective role in mast cell activation under chronic conditions. This study shows inhibitory effects of resveratrol on acute activation of mast cells by IL-33. It remains to be determined whether resveratrol affects not only acute, but also chronic responses to IL-33 in mast cells.

In summary, our findings show that resveratrol can inhibit IL-33/ST2– and IgE–mediated activation of mast cells principally by suppressing the MK2/3–PI3K/Akt axis. Thus, resveratrol may be applicable to broad ranges of allergic diseases.

## Supplementary information

Supplementary information
