# Supplementary material for: Resveratrol inhibits IL-33–mediated mast cell activation by targeting the MK2/3–PI3K/Akt axis
Source: Sci Rep. 2019 Dec 5;9:18423. doi: 10.1038/s41598-019-54878-5 (PMC6895112; doi:10.1038/s41598-019-54878-5)

Resveratrol inhibits IL-33–mediated mast cell activation by targeting the MK2/3–PI3K/Akt axis

Shotaro Nakajima,[1,5,6,7,†] Kayoko Ishimaru,[1,7] Anna Kobayashi,[1,2] Guannan Yu,[1] Yuki Nakamura,[1] Kyoko Oh-oka,[4] Katsue Suzuki-Inoue,[3] Koji Kono[5] and Atsuhito Nakao[1, ††]

[1]*Department of Immunology,* [2]*Department of Pediatrics,* [3]*Department of Clinical and Laboratory Medicine, Faculty of Medicine, University of Yamanashi, Yamanashi, Japan,*
[4]*Department of Immunology, Faculty of Medicine, Tukuba Advanced Research Alliance (TARA), University of Tsukuba, Tsukuba, Japan*
[5]*Department of Gastrointestinal Tract Surgery,* [6]*Department of Progressive DOHaD Research, Faculty of Medicine, Fukushima Medical University*
[7]*These authors equally contributed to this work*

[†, ††] **Corresponding author:** Department of Immunology, University of Yamanashi Faculty of Medicine, 1110 Shimokato, Chuo, Yamanashi 409-3898, Japan
Tel.:+81-55-273-6752; Fax.: +81-55-273-9542
E-mail: ipsho555@gmail.com (S. Nakajima), anakao@yamanashi.ac.jp (A. Nakao)
Present address (S. Nakajima): Department of Progressive DOHaD Research, Department of Gastrointestinal Tract Surgery, School of Medicine, Fukushima Medical University, 1 Hikariga-oka, Fukushima City, Fukushima 960-1295, Japan

Running title: Resveratrol inhibits IL-33–mediated mast cell activation.

Abbreviations: AMPK, AMP-activated protein kinase; ERK, extracellular-signal-regulated kinase; IKK, IκB kinase; IL-33, interleukin-33; JNK, c-Jun NH_2-terminal kinase; MAPK, mitogen-activated protein kinase; NF-κB, nuclear factor-κB; PI3K, phosphatidylinositol 3-kinase; PLC, phospholipase C; TAK1, transforming growth factor β–activated kinase 1; TNF, tumor necrosis factor

Supplementary Figure Legends

Supplementary Figure 1. Effect of resveratrol on cell survival and mRNA expression in BMMCs. (A) Flow cytometry analysis of Annexin V and 7-AAD in BMMCs treated with 10–100 μM resveratrol for 6 h (n=3). (B) WST assay of BMMCs treated with the indicated concentrations of resveratrol for 6 h (n=3). Data were represented as OD. (C) qPCR of IL-6, IL-13, and TNF-α in BMMCs stimulated with IL-33 for 1 h in the presence or absence of 25 μM resveratrol (n=3). (D) Flow cytometry analysis of FcεRI and c-kit in FSMCs. *P<0.05, **P<0.01, ****P<0.0001.

Supplementary Figure 2. Effect by resveratrol on estrogen receptor–mediated inhibition of cytokine expression by IL-33 in BMMCs. qPCR of IL-6, IL-13, and TNF-α in BMMCs stimulated with antigen for 1 h in the presence or absence of 25 μM resveratrol and/or ICI 182,780 (n=3). *P<0.05, ****P<0.0001.

Supplementary Figure 3. Effect by combination of resveratrol and PF on IL-33–

triggered cytokine expression in BMMCs. ELISA of IL-6 and IL-13 in BMMCs stimulated with IL-33 for 6 h in the presence or absence of 10 μM PF or combination of PF and 25 μM resveratrol (n=3). ***P<0.001, ****P<0.0001; n.s., not significant.

Supplementary Figure 4. Effect of resveratrol on SCF–mediated activation of Akt in BMMCs. (A) Western blot analysis of phospho–Akt, phospho–p70S6K, and phospho–p38 in BMMCs stimulated with 1 ng/ml IL-33 or 100 ng/ml SCF for 15 min in the presence or absence of 25 μM resveratrol. The level of β-actin is shown at the bottom as a loading control.

Supplementary Figure 5. Effect of resveratrol on IgE–mediated mast cell activation. (A) Intracellular Ca^{2+} mobilization in BMMCs stimulated with antigen for up to 180 sec in the presence or absence of resveratrol (n=3). (B) Flow cytometry analysis of CD63 in BMMCs treated with antigen for 40 min in the presence or absence of 25 μM resveratrol (n=3). (C) Release of β-hexosaminidase from BMMCs stimulated with antigen in the presence or absence of resveratrol (n=3). (D) ELISA of IL-6, IL-13,

and TNF-α in BMMCs treated with antigen for 6 h in the presence or absence of resveratrol (n=3). (E) Passive cutaneous anaphylaxis in dorsal skin of mice treated orally with 10 mg/kg resveratrol or 2% cyclodextrin in phosphate-buffered saline (PBS) (as a control) (n=4). The bottom panel shows the density of blue-stained in the upper panel. (F) Serum histamine levels after passive cutaneous anaphylaxis reaction in mice treated orally with 10 mg/kg resveratrol (n=3–4). *P<0.05, **P<0.01, ***P<0.001, ****P<0.0001.

Supplementary Figure 6. Effect of resveratrol on basal and antigen-induced phosphorylation of AMPK in BMMCs. (A) Western blot analysis of phospho-AMPK in BMMCs treated with 25 μM resveratrol or 0.5 mM AICAR for up to 12 h. The level of β-actin is shown at the bottom as a loading control. (B) Western blot analysis of phospho–AMPK, phospho–Akt, and phospho–p38 in BMMCs stimulated with antigen for up to 10 min in the presence or absence of resveratrol or AICAR. The level of β-actin is shown at the bottom as a loading control.

Supplementary Figure 7. Full-length blots shown in figures and supplementary figures.

Supplementary Figure 1

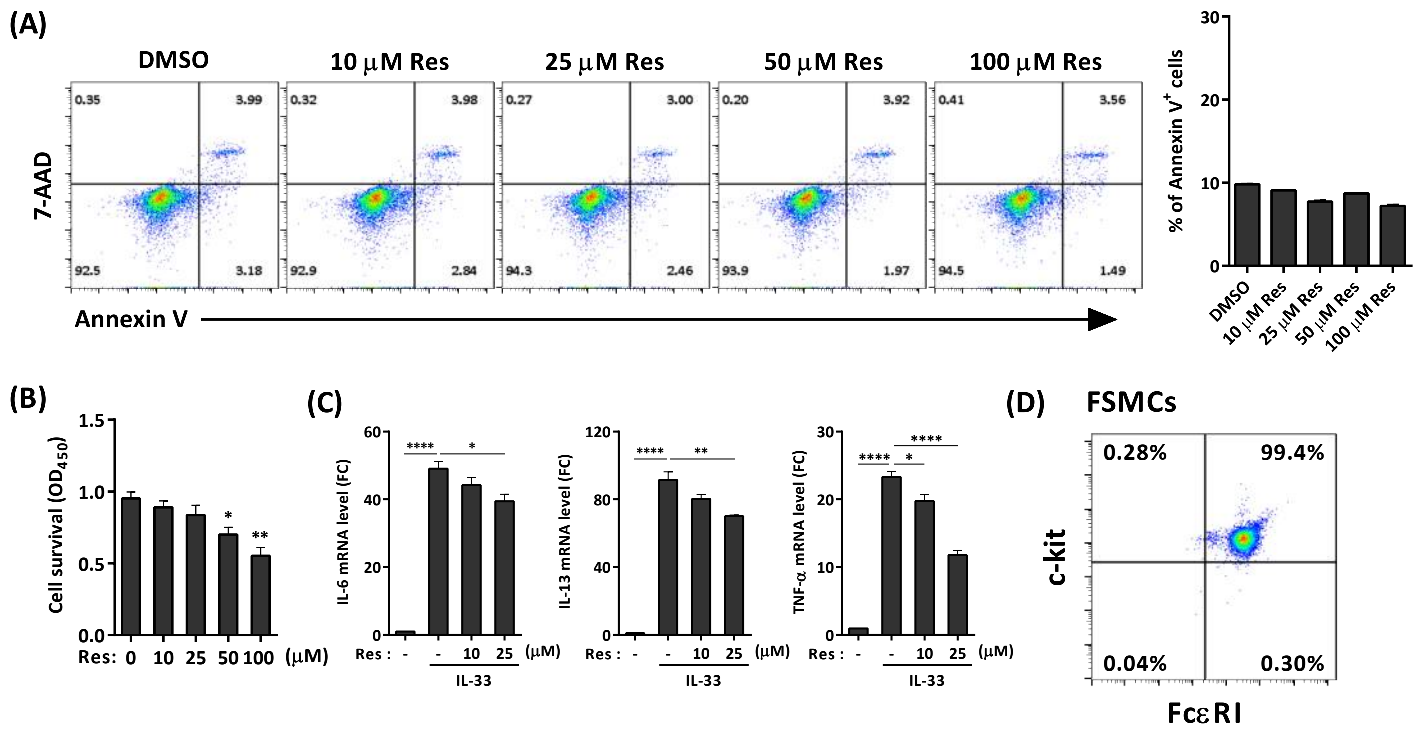

Supplementary Figure 2

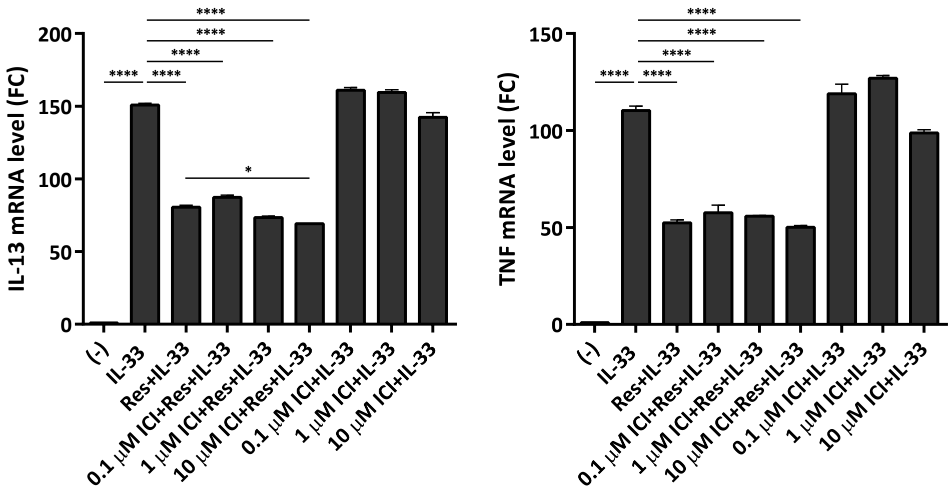

Supplementary Figure 3

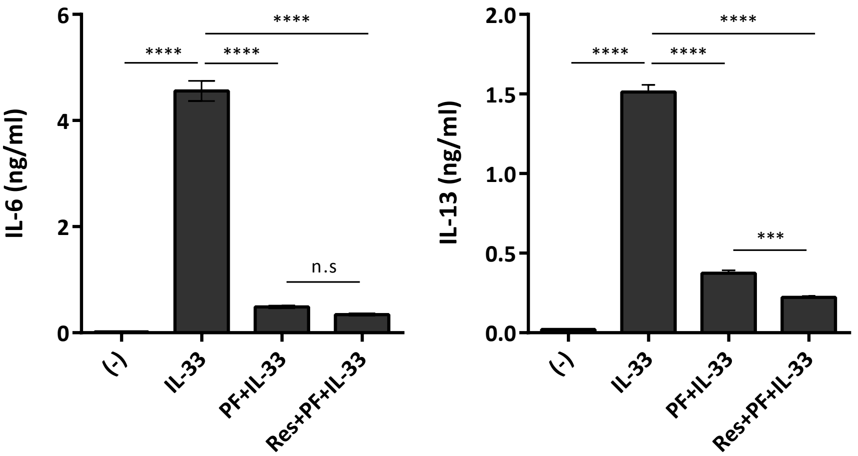

Supplementary Figure 4

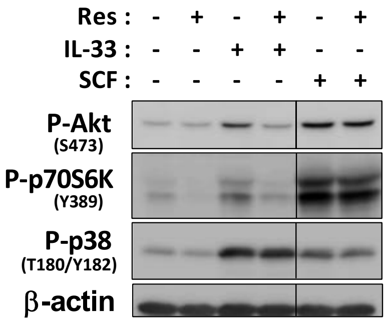

Supplementary Figure 5

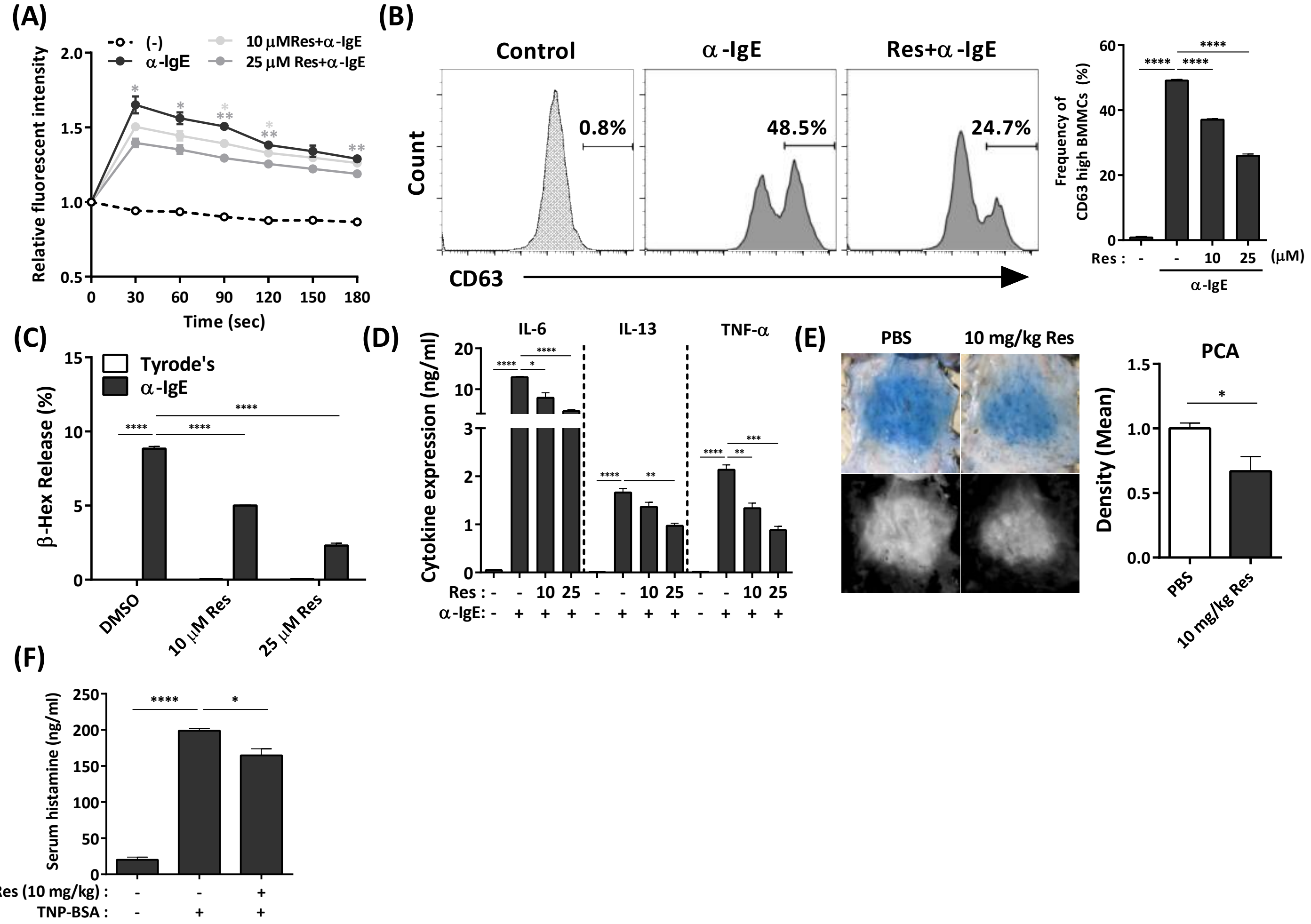

Supplementary Figure 6

(A)

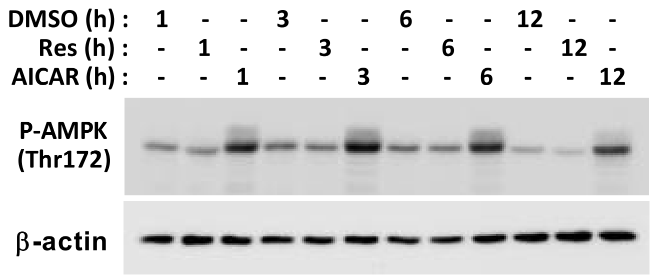

(B)

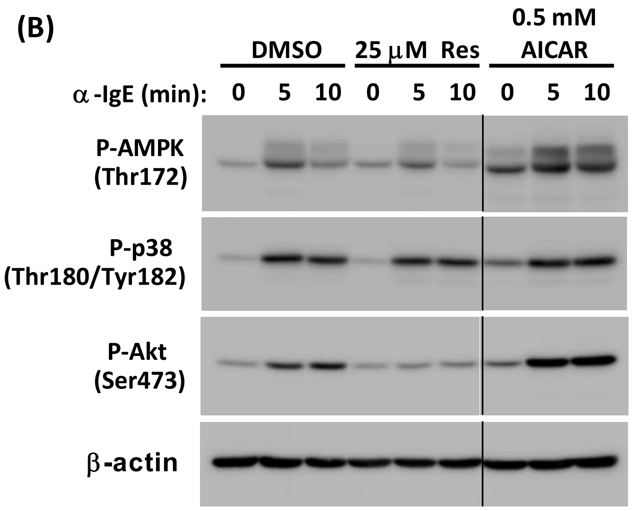

Supplementary Figure 7-1

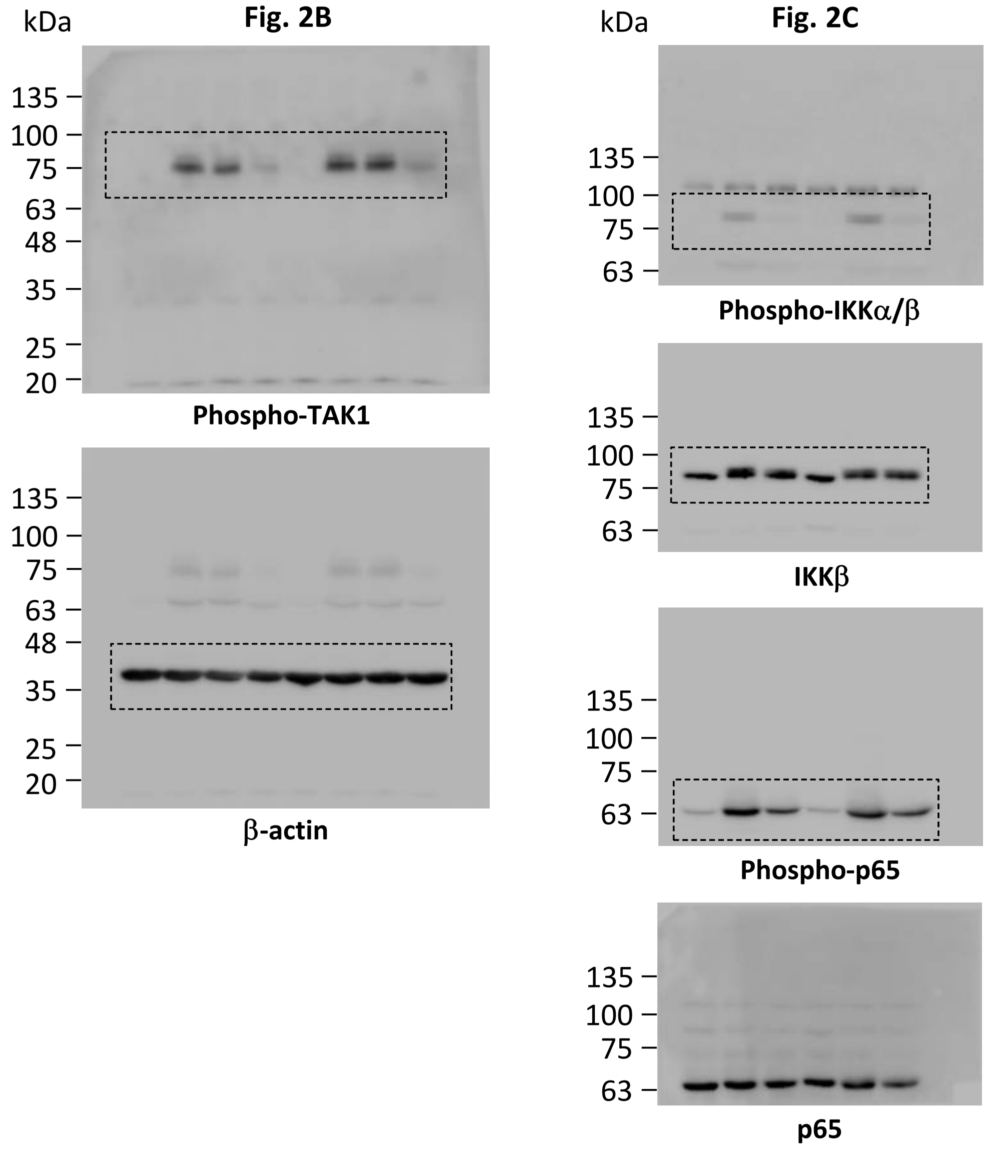

Supplementary Figure 7-2

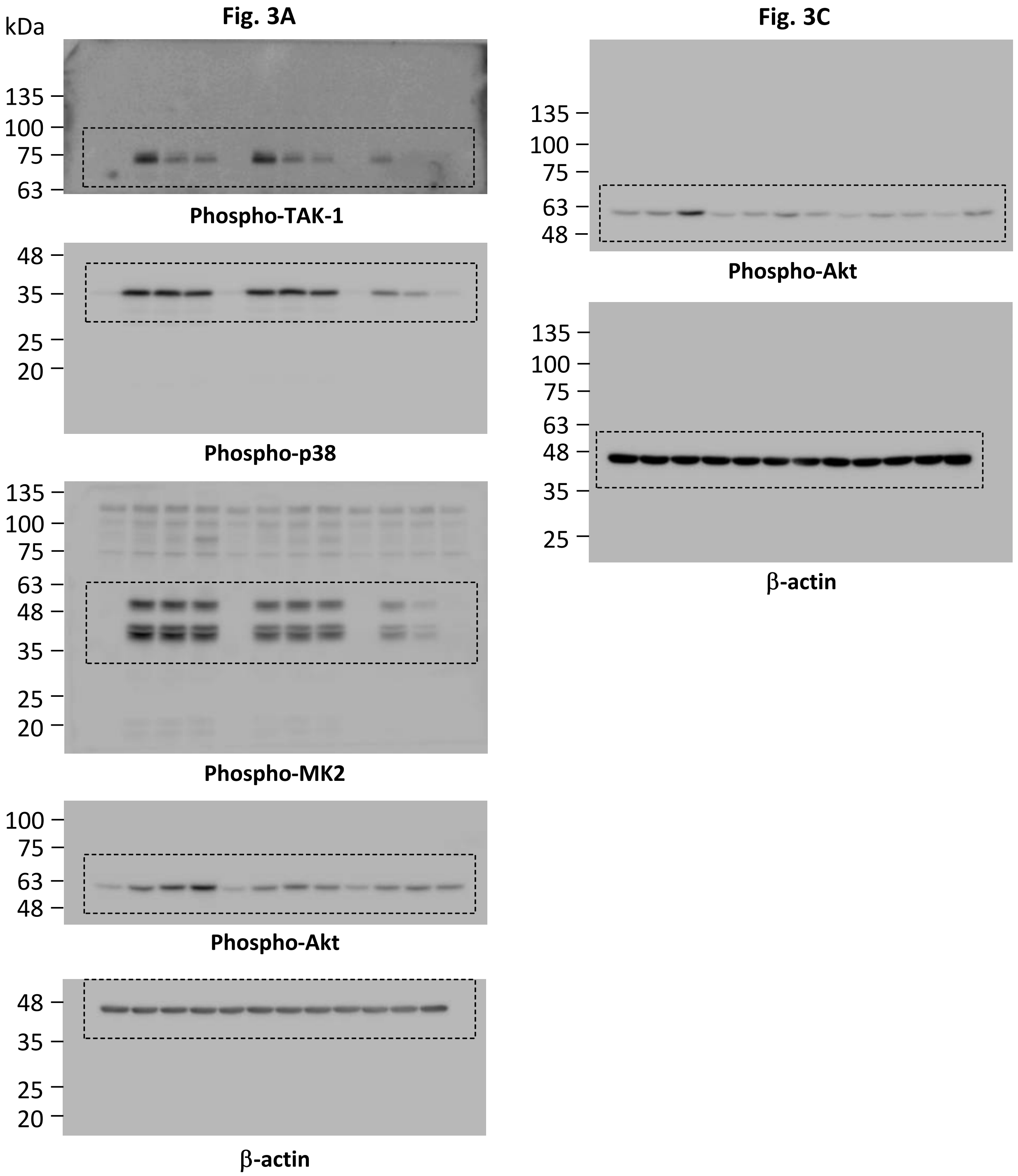

Supplementary Figure 7-3

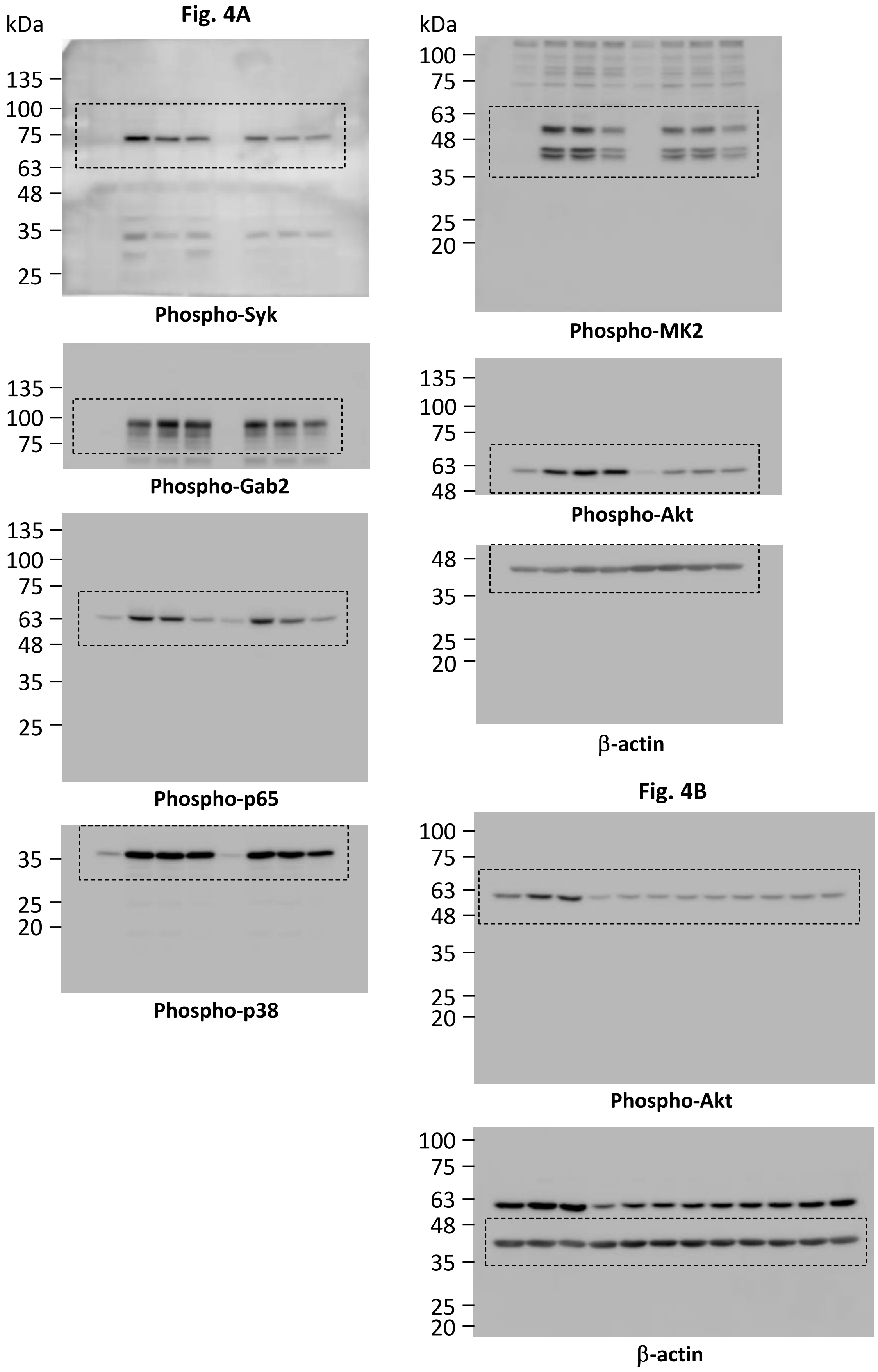

Supplementary Figure 7-4

Fig. S3

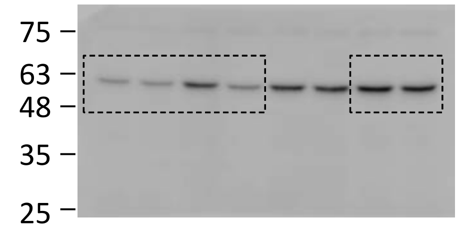

Phospho-Akt

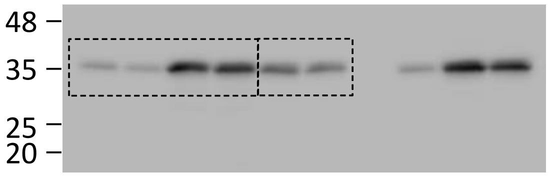

Phospho-p38

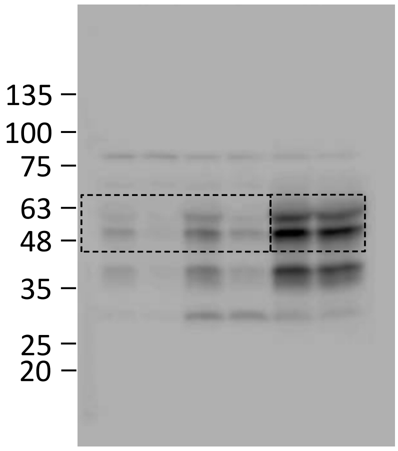

Phospho-p70S6K

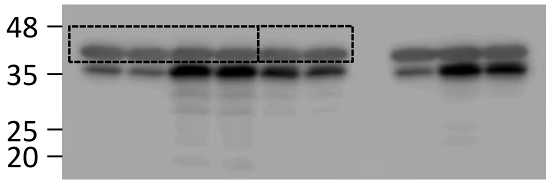

β-actin

Fig. S5A

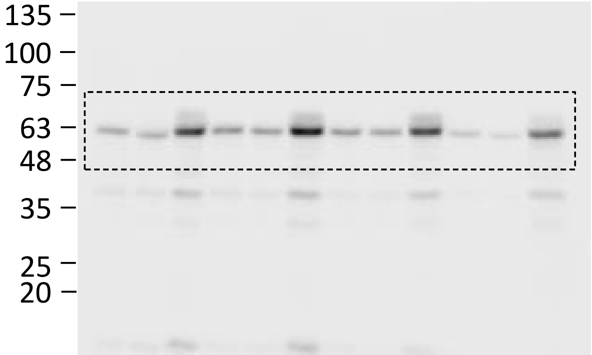

Phospho-AMPK

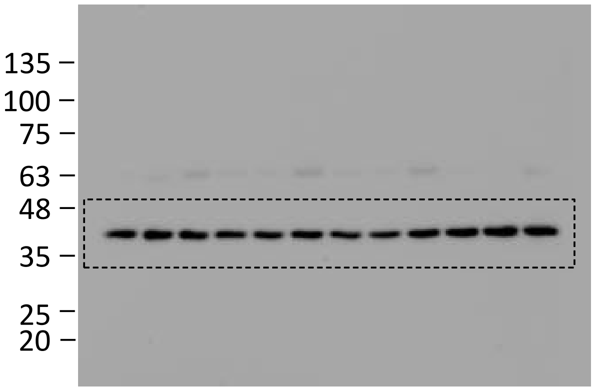

β-actin

Supplementary Figure 7-5

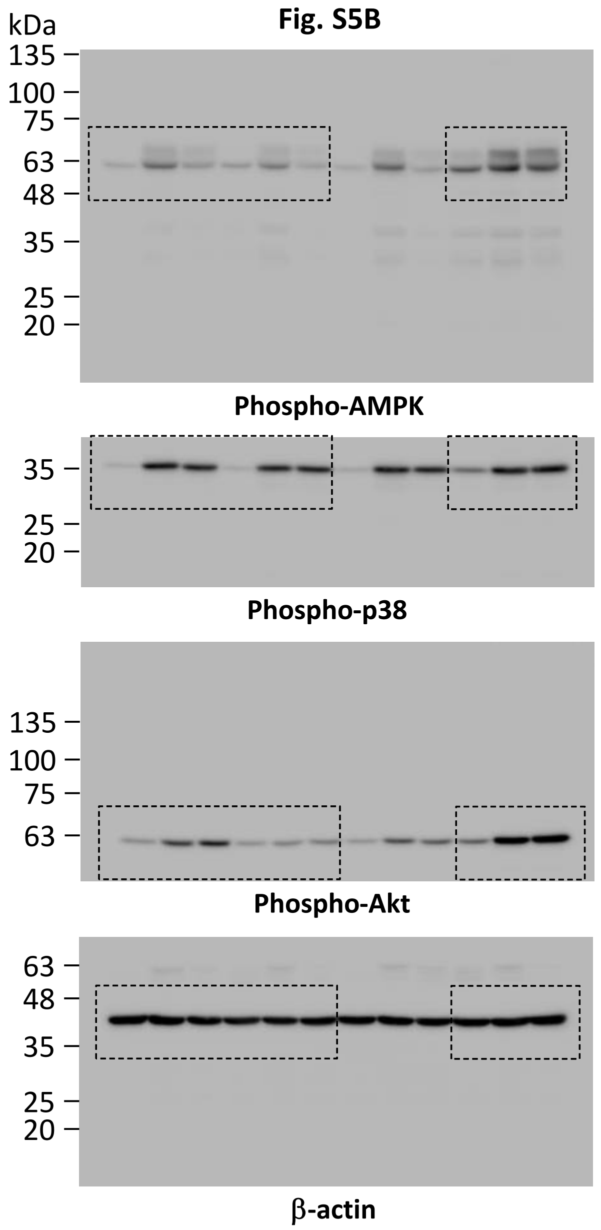

Supplement: Supplementary file 1 — Supplementary information [file 41598_2019_54878_MOESM1_ESM.pdf]
